# Effects of rare earth elements on bacteria in rhizosphere, root, phyllosphere and leaf of soil–rice ecosystem

**DOI:** 10.1038/s41598-022-06003-2

**Published:** 2022-02-08

**Authors:** Xinzhuan Zhang, Zhongjun Hu, Huahua Pan, Yijun Bai, Ying Hu, Shulan Jin

**Affiliations:** 1 Shangrao Normal University, No.401, Zhimin Road, Xinzhou District, Shangrao, 334000 China; 2 Research Center for Eco-Environmental Sciences, Chinese Academy of Sciences, No.18, Shuangqing Road, Haidian District, Beijing, 100085 China

**Keywords:** Soil microbiology, Plant stress responses

## Abstract

The effects of rare earth mining on rice biomass, rare earth element (REE) content and bacterial community structure was studied through pot experiment. The research shows that the REE content in rice roots, shoots and grains was significantly positive correlated with that in soil, and the dry weight of rice roots, shoots and grains was highly correlated with soil physical and chemical properties, nutrient elements and REE contents; The exploitation of rare earth minerals inhibited a-diversity of endophytic bacteria in rhizosphere, root, phyllosphere and leaf of rice, significantly reduced the abundance index, OTU number, Chao, Ace index and also significantly reduced the diversity index–Shannon index, and also reduced uniformity index: Pielou’s evenness index, which caused β-diversity of bacteria to be quite different. The exploitation of rare earth minerals reduces the diversity of bacteria, but forms dominant bacteria, such as *Burkholderia*, *Bacillus*, *Buttiauxella*, *Acinetobacter*, *Bradyrhizobium*, *Candida koribacter*, which can degrade the pollutants formed by exploitation of rare earth minerals, alleviate the compound pollution of rare earth and ammonia nitrogen, and also has the function of fixing nitrogen and resisting rare earth stress; The content of soil available phosphorus in no-mining area is lower, and the dominant bacteria of Pantoea formed in such soil, which has the function of improving soil phosphorus availability. Rare earth elements and physical and chemical properties of soil affect the community structure of bacteria in rhizosphere and phyllosphere of rice, promote the parallel movement of some bacteria in rhizosphere, root, phyllosphere and leaf of rice, promote the construction of community structure of bacteria in rhizosphere and phyllosphere of rice, give full play to the growth promoting function of Endophytes, and promote the growth of rice. The results showed that the exploitation of rare earth minerals has formed the dominant endophytic bacteria of rice and ensured the yield of rice in the mining area, however, the mining of mineral resources causes the compound pollution of rare earth and ammonia nitrogen, which makes REE content of rice in mining area significantly higher than that in non-mining area, and the excessive rare earth element may enter the human body through the food chain and affect human health, so the food security in the REE mining area deserves more attention.

## Introduction

China is a major producer, exporter and consumer of rare earth resources in the world. It was reported that China owns about 50 percent of the world's rare earth resources and provides about 90 percent of the global supply of rare earth^1^. South China, with Ganzhou City of Jiangxi Province as the center, is an important distribution area of ion-type rare earth resources in the world, and its reserves account for about 70% of the world's total reserves^2^. The illegal and disorderly exploitation of rare earth resources caused large amount of rare earth elements to enter the soil and water environment, which affects the growth of plants, animals and microorganisms in the environment, thus changes the ecological environment and affects human health through the food chain^3,4^. Rice is the main grain of south China, and the yield and quality of rice are closely related to the ecological environment of cultivated land. Microorganism is the most sensitive indicator of farmland ecological environment. The microorganism is affected by soil physical and chemical properties, plant species/genotypes, and also affected by heavy metal pollution^5,6^.

With the extensive use of rare earth resources in many industries, the world's demand for rare earth resources increased from 136,000 tons in 2017 to 151,000 tons in 2020^7^. The impact of rare earth mining and smelting on the environment has attracted much attention. It is reported that the REE content in soil of rare earth mining area in South China is 396–2314 mg/kg^8^, which is much higher than the average REE content in soil of China. The concentration of rare earth in water is 1000 times^9^ higher than that in ordinary fresh water. Rare earth elements in ecosystems not only affect plants and animals, but also affect the growth, reproduction, migration and colonization of microorganisms^10,11^. Rare earth elements affect the growth indexes of plants, including the biomass of above-ground and underground parts. It affects the index of plant resistance system, such as plant enzyme activity, etc. It also affect the REE content in plants^12,13^. The appropriate concentration of rare earth elements can promote the growth and reproduction of microorganism and the increase of metabolites, while high concentration of rare earth can inhibit growth and reproduction of microorganism^14^. Chu et al.^15^ found that La^3+^ has strong toxic effect on bacteria, actinomycetes, fungi and other microorganisms in the microflora of red soil. He et al.^16^ found that rare earth elements had strong inhibitory effect on *Escherichia coli*, *Staphylococcus aureus* and *Candida albicans*. Wang et al.^17^ found that the changes of soil REE content and physical and chemical properties significantly affected the diversity and abundance of soil bacteria. Wang^18^ found that the microbial biomass in the areas with serious accumulation of rare earth was inhibited, the bacterial diversity in the non-rare earth mining area was higher than that in the mining area. The REE content is the main factor affecting the microbial structure. Rare earth elements are very important for *methoxybacteria* in extremely acidic environment^19–21^.

After rare earth elements enter the soil, they directly affect the microorganisms in the soil–plant ecosystem, and also indirectly affect the microorganisms by influencing the physical and chemical properties of soil and plant bio-chemical system. Microorganisms carry out the processes of oxidation, nitrification, ammoniation, nitrogen fixation and vulcanization in soil to promote the decomposition of soil organic matter and the transformation of nutrients. Endophytes have significant effects on plants, e.g. endophytic bacteria has nitrogen fixation effect on rice. The common endophytic azotobacters are *Pseudomonas*, *Spirulina*, *Enterobacteriaceae* and *Bacillus,* they reduce the free N_2_ in the atmosphere into the available NH_4_^+^ by nitrogenase^22,23^. The endogenous bacteria can secrete indoleacetic acid, which promote the cell division and root growth of plants, assist host rice to improve the absorption efficiency of N, P and K, improve the production of gibberellin and salicylic acid in rice, thus promote the growth of rice^24–26^. Endophytic bacteria can help to resist sheath blight, and the mechanism of rice endophytes assisting host rice disease resistance mainly includes secretion of antibiotics, hyperparasitism, niche competition and induction of rice self-resistance^27,28^. To resist abiotic stress, endophytic bacteria can help plants polluted by heavy metals obtain bioremediation, e.g. Zhou et al.^29^ found that endophytic bacteria *Stenotrophomonas maltophilia* R5-5 significantly reduced the Cd content in rice plants by down regulating the gene expression of cadmium absorption and transport proteins in rice and changing the community structure of endophytic bacteria, and endophytic bacteria can effectively protect plants from the toxicity of many heavy metals (Cd, Cu, Cr, Mn, Ni, Pb and Zn)^30^.

At present, the research on the effect of endophytes on rice is mainly carried out in uncontaminated habitats, but the research carried out in rare earth polluted habitats has not been reported. In this study, the soil of typical REE mining area in Ganzhou City of Jiangxi Province was taken as the research object, and the soil of non-mining area was taken as the control check (CK). The effects of rare earth elements on bacteria in rhizosphere, root, phyllosphere and leaf of soil–rice ecosystem were discussed, which has scientific value and practical significance.

## Results

### Effects of mineral exploitation on soil physical and chemical properties, REE content, rice biomass and REE content

As can be seen from Table 1 that the contents of rare earth elements, ammonia nitrogen, nitrate nitrogen, total P and available P in soil of H and M at both tillering stage and maturity stage of rice are significantly higher than those in soil of L, which was taken as CK from paddy field in non-mining area of Choukou village, Xinzhou District of Shangrao City, Jiangxi Province, and the pH value is lower than that of in soil of L. The results show that the mining of rare earth minerals can improve the REE content in soil and affect the physical and chemical properties of soil.

**Table 1:** Pearson correlation of rare earth element content, soil physical and chemical properties and α-diversity of rice bacteria at tillering stage.

	Shannon	Observed_richness	Pielou_evenness	Chao.value
Soil REE content	− 1.000*	− 0.812	− 0.991	0.521
Root REE content	− 0.806	− 0.28	− 0.865	0.936
Shoot REE content	− 0.815	− 0.294	− 0.872	0.931
Total N	− 0.327	− 0.834	− 0.224	− 0.613
Ammonia N	− 0.987	− 0.882	− 0.964	0.404
Nitrate nitrogen	− 0.794	− 1.000**	− 0.724	− 0.075
Total P	− 0.559	− 0.948	− 0.467	− 0.388
Available P	− 0.47	− 0.91	− 0.373	− 0.482
Available K	0.261	− 0.38	0.363	− 0.951
Organic matter	0.272	− 0.369	0.374	− 0.955
pH	0.949	0.945	0.909	− 0.254

**At 0.01 level (two tailed), the correlation was significant, *At 0.05 level (two tailed), the correlation was significant, n = 3.

**Figure 1:**
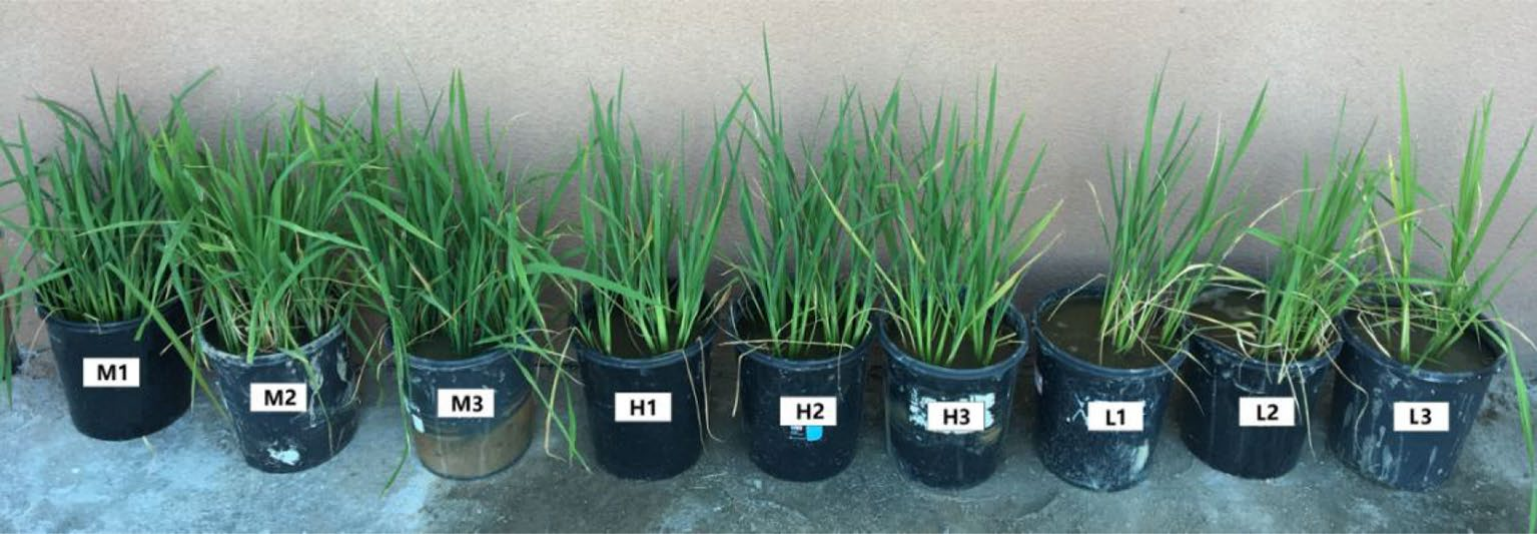
Growth of rice at tillering stage. H1–H3 are soil samples in the mining area with high REE content, M1–M3 are soil samples in the mining area with medium REE content, L1–L3 is soil sample in the non-mining area.

**Figure 2:**
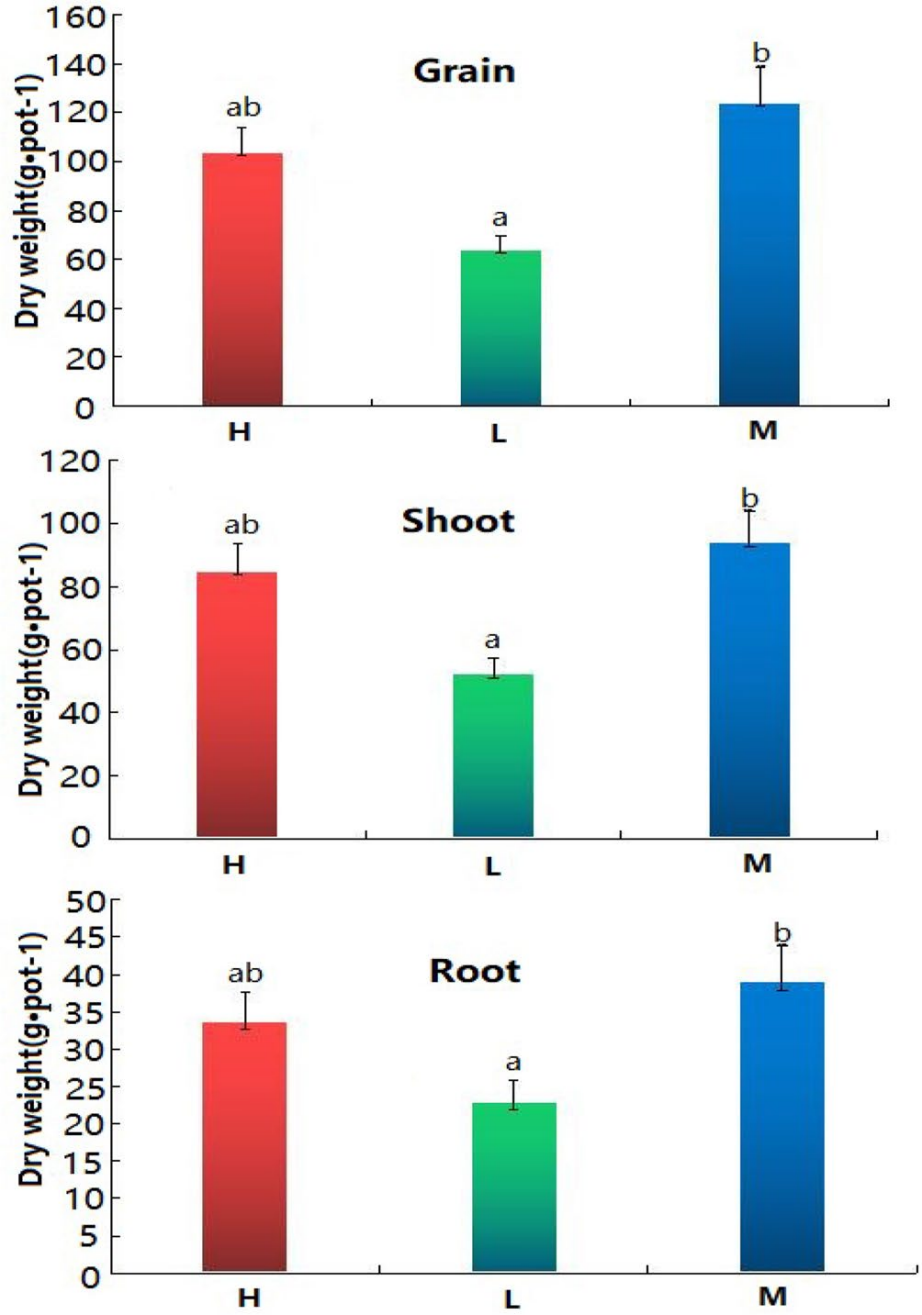
Dry weight of grain, shoot and root of rice. Different letters indicate significant differences between potted rice in different soils.

As can be seen from Fig. 1 that the tillering shoots of rice cultivated in M soil are the most at tillering stage, and the rice grew best; The rice cultivated in soil H has more tillers and leaves and grew better; The tillering shoots of rice grew in soil L soil were fewer and the growth vigor of rice was worse than others. Figure 2 shows that the weight m of root, shoot and grain of rice is the largest in soil M, with an average of 38.9 g, 93.8 g and 123.8 g respectively, followed by soil H, with an average of 33.7 g, 84.7 g and 103.7 g respectively, and then soil L, with an average of 22.8 g, 52.1 g and 63.7 g respectively. The weight of rice root, shoot and grain of M was 1.15, 1.11 and 1.19 times that of H, and 1.71, 1.80 and 1.94 times that of L, respectively.

**Figure 3:**
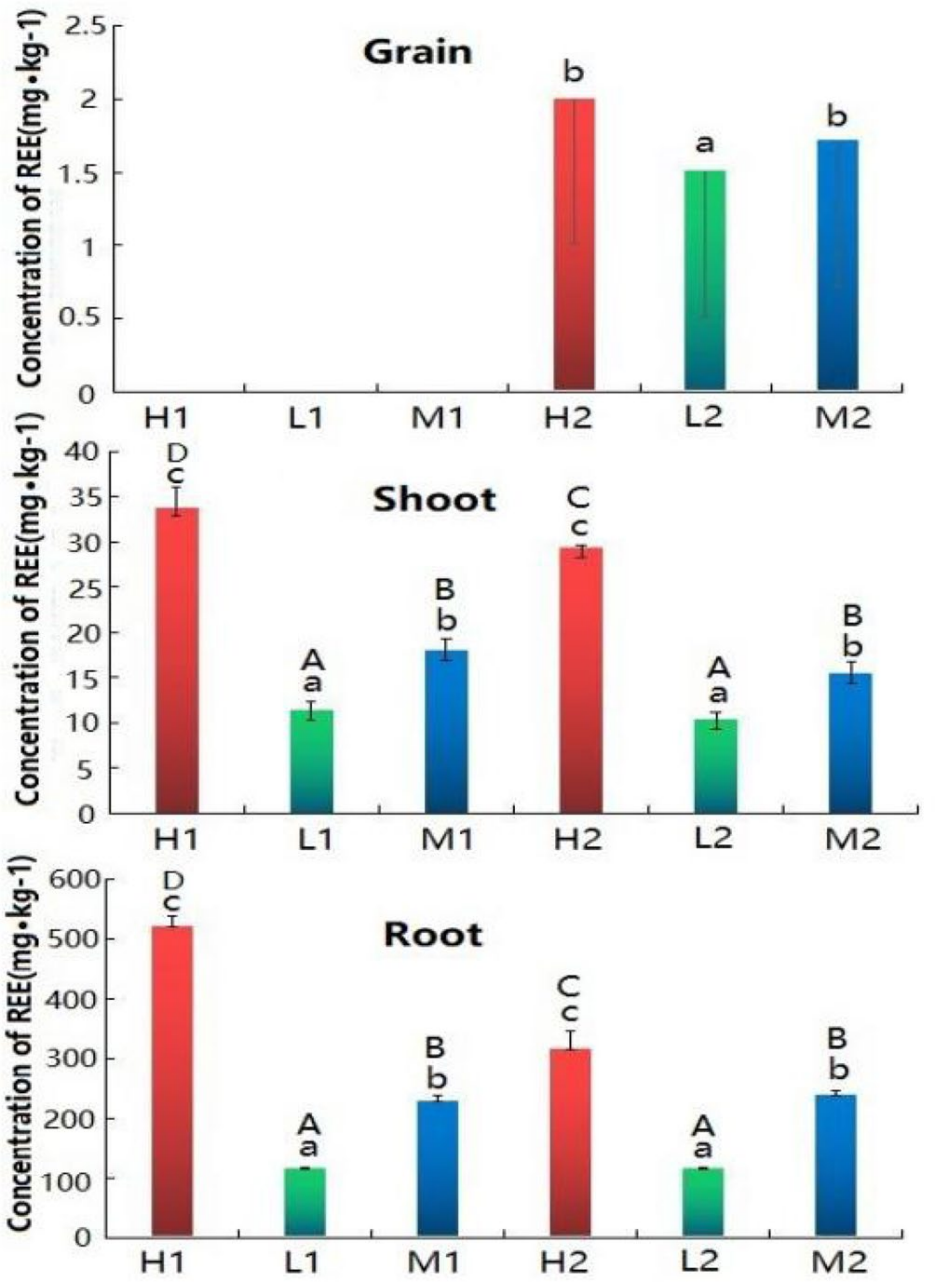
Content of rare earth elements in shoots and roots of rice at tillering stage and grains, shoots and roots at maturity stage. Different letters indicate significant differences between pot experiment rice in different soil (lowercase letters indicate significant differences in the same stage, capital letters indicate significant differences between the two stages), P < 0.05.

It can be seen from Fig. 3 that the REE contents in different parts of rice at tillering stage and maturity stage are significantly positively correlated with the REE contents in soil. The REE contents in roots, shoots and grains of rice cultivated in soil H were the highest, followed by those cultivated in soil M and L. At tillering stage, the REE content in rice roots, shoots of H were 2.28 and 1.89 times that of M, 4.53 and 2.98 times that of L, respectively. At maturity stage, the REE content in rice roots, shoots and grains of H were 1.32, 1.91 and 1.17 times that of M, and 2.76, 2.83 and 1.33 times that of L, respectively.

Figure 3 shows the REE contents in different parts of rice at tillering stage and maturity stage are significantly positively correlated with the REE contents in soil. The REE contents in rice roots, shoots and grains cultivated in soil H were the highest, followed by those cultivated in soil of M and L. At tillering stage, the REE contents in roots, shoots of rice cultivated in soil H were 2.28 and 1.89 times of those in soil M, 4.53 and 2.98 times of those in soil L, respectively; At maturity stage, the REE contents in roots, shoots and grains of rice cultivated in soil H were 1.32, 1.91 and 1.17 times that of rice cultivated in soil M, and 2.76, 2.83 and 1.33 times that of rice cultivated in soil L, respectively.

### Effects of soil REE content and physicochemical properties on microbial diversity in rhizosphere, root, phyllosphere and leaf of rice

**Figure 4:**
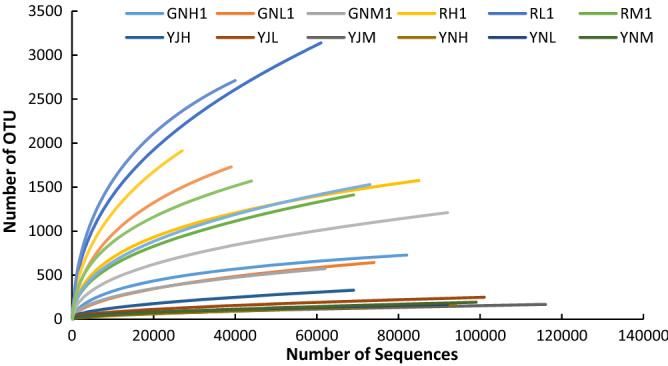
Dilution curve of bacterial 16S gene sequence.

REE contents of soil can affect bacteria α-diversity, including abundance index: OTU number, Chao, Ace; Diversity index: Shannon Weiner index, Evenness index: Pielou's evenness index, etc. GN (H1, M1, L1), R (H1, M1, L1), YJ (H, M, L) and YN (H, M, L) in Fig. 4 respectively represent the bacteria in the root, rhizosphere, phyllosphere and leaf of rice at tillering stage, GN (H2, M2, L2) and R (H2, M2, L2) respectively represent the bacteria in the root and rhizosphere of rice at maturity stage, the same below. It can be seen from Fig. 4 that the number of OTU is affected by REE content of soil. The number of OTU in rhizosphere, root, phyllosphere and leaf of rice cultivated in soil of H and M of rare earth mining area is mostly lower than that of rice cultivated in soil L of non-rare earth mining area. At tillering stage, the difference of OTU number among three different soil samples was the largest. The OTU number of sample L on the same read number was the largest, followed by that of sample H and M. When the number of sequencing reads was 61,000, the OTU number of sample L was 2.23 and 2.34 times that of sample H and M, respectively. The OTU number of rhizosphere bacteria in sample L was the largest, followed by sample H and M. When the number of sequencing reads was 27,000, the OTU number of rhizosphere bacteria in sample L was 1.24 and 1.81 times that of sample H and M respectively. When the number of sequencing reads was 39,000, the OTU number of rhizosphere bacteria in sample L sample was 1.47 and 2.07 times that of sample H and M, respectively.

**Figure 5:**
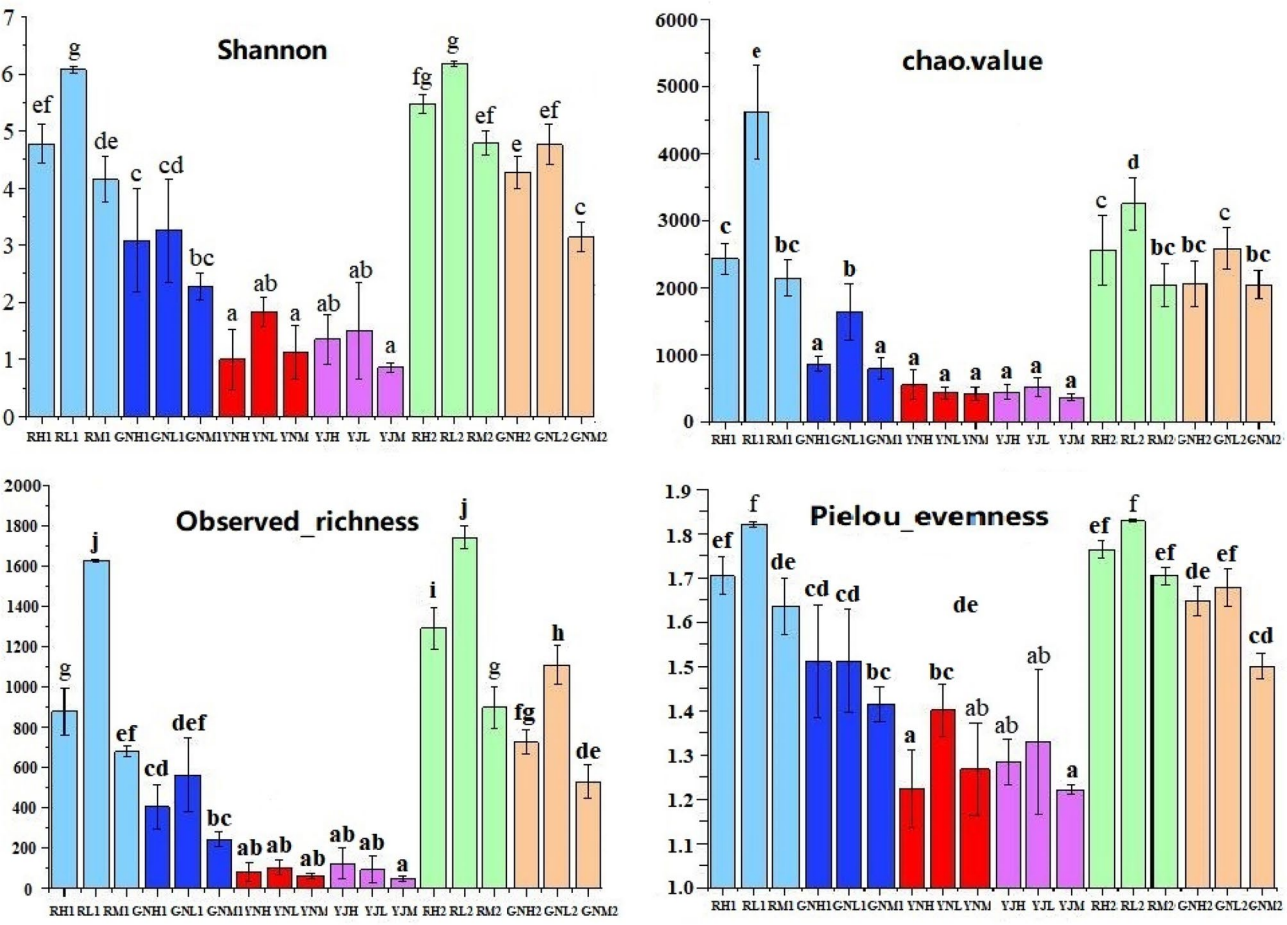
α-diversity of bacteria in root, rhizosphere, leaf and phyllosphere of rice at tillering and maturity stages. Notes: Different letters indicate the significant difference among bacteria of different soil pot-experiment rice (small letters indicate the significant difference in the same stage, capital letters indicate the significant difference between the two stages), P < 0.05.

As can be seen from the Fig. 5 that the Shannon index of rhizosphere, root, phyllosphere and leaf of rice cultivated in soil H and M of mining area was lower than that cultivated in soil L of non-mining area, especially the Shannon index of rhizosphere and endophytic phyllosphere of rice cultivated in soil H and M in tillering stage was significantly lower than that cultivated in soil L, which were only 0.55 and 0.61 times that of soil L, respectively. The Chao1 index of bacteria in rice rhizosphere, root, phyllosphere of group H and M was lower than that of group L at tillering stage and maturity stage, and the significant difference was that the bacteria in rice rhizosphere and root of group H and M at tillering stage were 0.53, 0.46, 0.52 and 0.48 times that of group L, respectively. The richness of most samples in group H and M was lower than that in group L. The richness of bacteria in rice rhizosphere and endophytic of group H and M was significantly lower than that of group L at tillering stage and maturity stage, which were 0.54, 0.42, 0.72, 0.43, 0.74, 0.52, 0.65 and 0.48 times that of group L, respectively. Mining reduced the Pielou's evenness index of bacteria, and the most important factor was the leaf bacteria at tillering stage. The index of group H and M was only 0.56 and 0.67 times that of group L, respectively.

**Figure 6:**
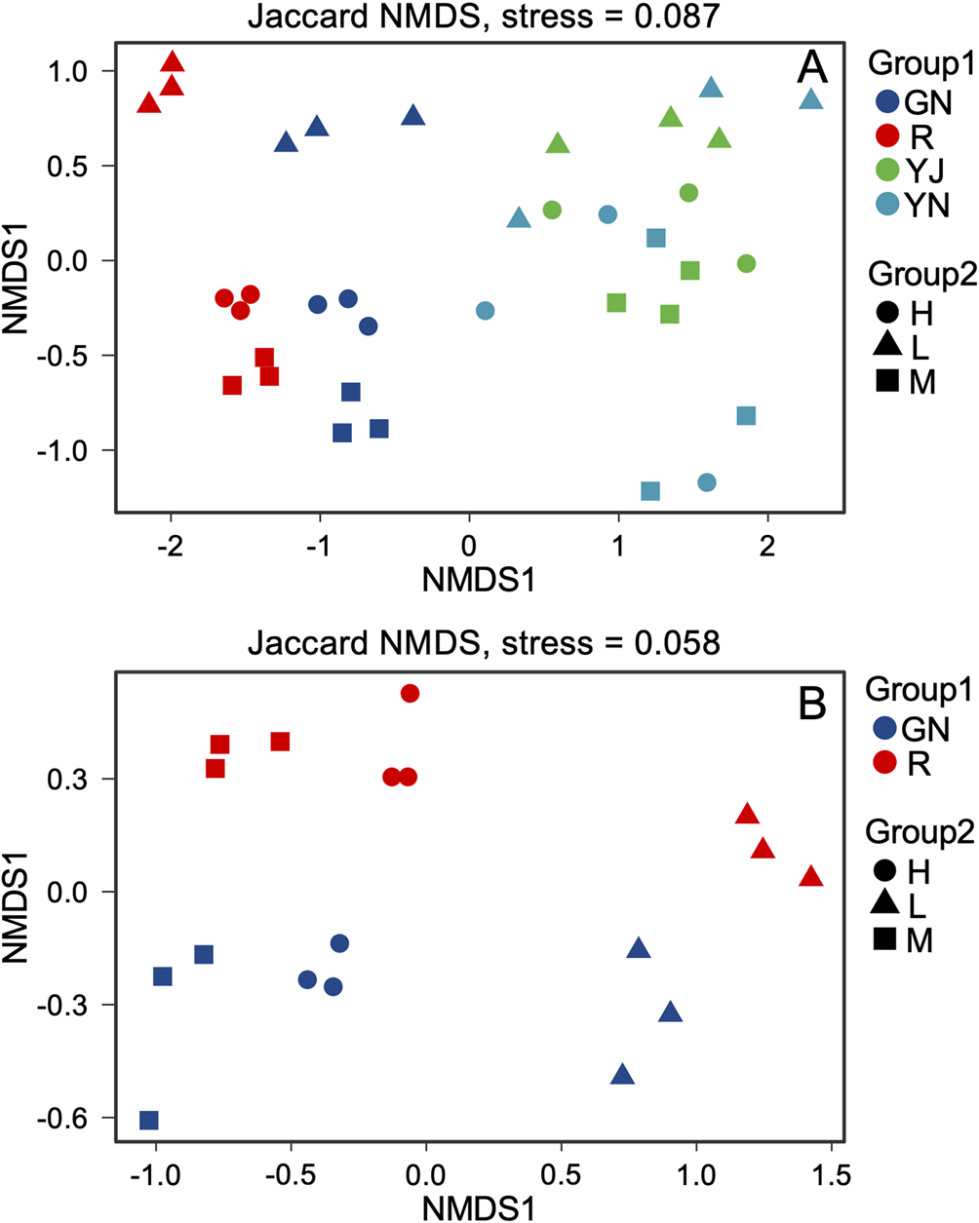
β-diversity of bacterial community structure of pot-experiment rice in different soils based on NMDS analysis.

A and B are the results of NMDS analysis at tillering stage and maturity stage respectively in Fig. 6. Figure 6 shows that the bacteria in different parts of rice cultivated in the same soil are highly dispersed, that is, the bacteria in rhizosphere, root, phyllosphere and leaf are dispersed in both tillering stage and maturity stage; The bacteria in the same part of rice cultivated in soils with different REE contents were also dispersed, especially the difference between soil H, M in mining area and soil L in non mining area was larger. Figure 6 shows that the stress values of A and B are 0.087 and 0.058, respectively, which are both less than 0.1, indicating that the difference of NMDS Bray Curtis is significant. The effect of rare earth mining on β-diversity of bacteria is obvious.

### Effects of soil REE content and physicochemical properties on bacterial community structure in rhizosphere, root, phyllosphere and leaf of rice

**Figure 7:**
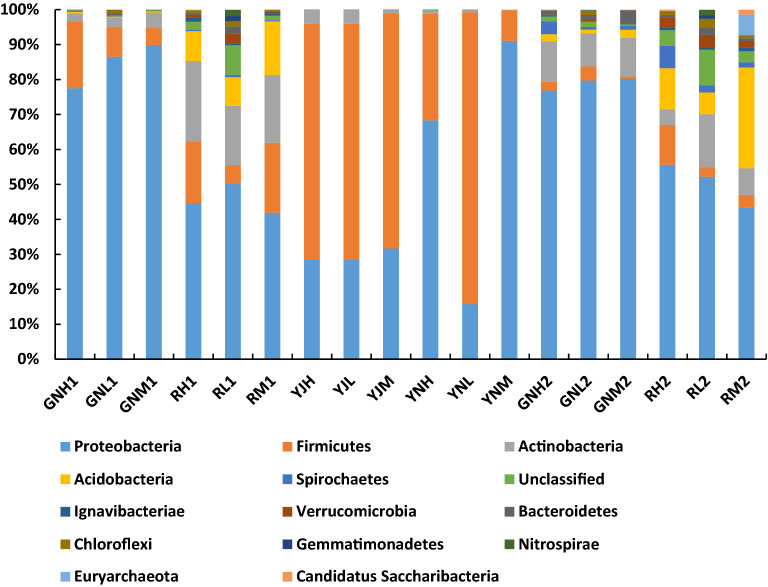
Bar chart of the phylum level.

Figure 7 has shown that Proteobacteria, Firmicutes, Actinobacteria, Acidobacteria, etc. exist in rhizosphere, root, phyllosphere and leaf of rice at tillering stage and in rhizosphere, root of rice at maturity stage. Rare earth mining has significant effect on Proteobacteria of rice at tillering stage. The relative abundance of this phyla in group H and M is much higher than that in group L, which is 4.33 and 5.77 times that in group L, respectively; The relative abundance of Firmicutes in rhizosphere of group H and M was significantly higher than that of group L at tillering stage, and the number of bacteria in leaves of group H and M was significantly lower than that of group L. the former was 3.47 and 3.95 times that of group L, while the latter was 0.37 and 0.11 times that of group L, respectively; The relative abundance of group H and M was 0.61 and 0.14 times higher than that of group L, respectively; The relative abundance of group H and M was 4.40 and 1.44 times higher than that of group L, respectively. The relative abundance of Actinobacteria in rhizosphere of group H and M was 0.29 and 0.50 times that of group L, respectively. The relative abundance of Actinobacteria in rhizosphere and root of group H and M was 1.81 and 2.15 times that of group L, and 1.88 and 4.62 times that of group L, respectively.

**Figure 8:**
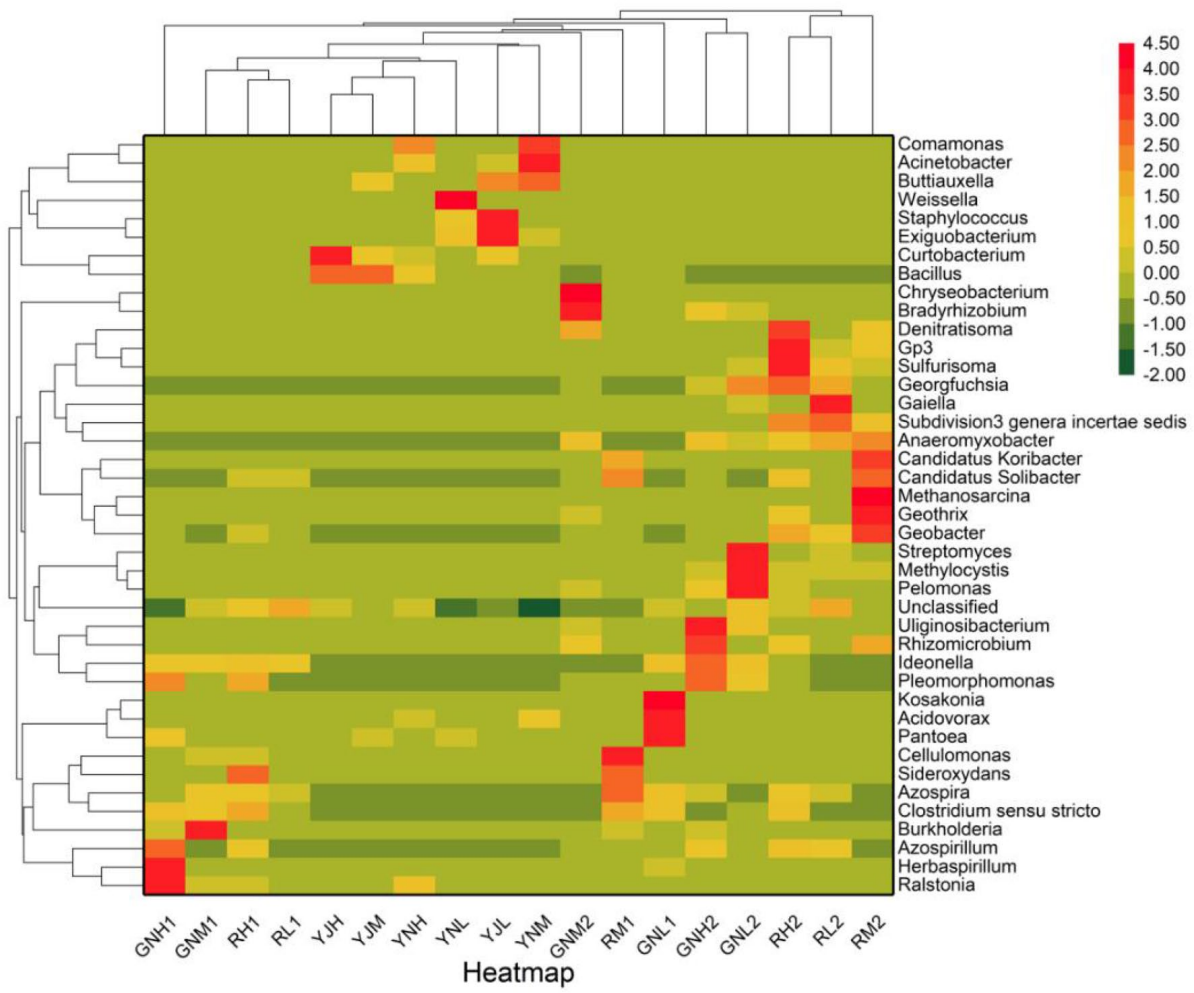
Heatmap of genus level.

As can be seen from Fig. 8 that the mining of rare earth significantly improved the relative abundance of *Ralstonia* and *Burkholderia* in rice roots at tillering stage, and reduced the relative abundance of *Pantoea*, *Acidovorax*, *Azospira*, *Buttiauxella* and *Kosakonia* in roots. The relative abundance of *Ralstonia* in roots of group H and M was 63.42 and 7.5 times that of group L, and the relative abundance of *Pantoea* in roots of group L was 3.29 and 8474 times that of group H and M, respectively. The relative abundances of *Burkholderia* in group M were 9.23 and 87.71 times higher than those in group L, respectively. The relative abundances of *Bacillus*, *Clostridium *sensu stricto, *Azospira*, *Sideroxydans*, *Cellulomonas* and *Candida solibacter* in rice rhizosphere increased, and the relative abundances of *Bacillus* and *Pantoea* in rice phyllosphere also increased, while the relative abundances of *Exiguobacterium*, *Buttiauxella*, *Acinetobacter* and *Staphylococcus* decreased. The relative abundance of *Bacillus* of group M was 8.01 and 8.28 times that of group L, respectively. The relative abundance of *Exiguobacterium* and *Buttiauxella* of group L was 44.41, 194.48, 100.83 and 2.11 times that of group H and M, respectively. The relative abundance of *Acinetobacter* and *Comamonas* in leaves increased, and the relative abundance of *Pantoea*, *Exiguobacterium*, *Staphylococcus* and *Weissella* in group L decreased. The relative abundances of *Pantoea*, *Exiguobacterium*, *Weissella* and *Staphylococcus* in leaves of group L were 4.72, 55.03, 61.81, 2.88, 45.93, 454.58, 33.00 and 31.43 times that of those in leaves of group H and M, respectively. In addition, *Herbaspirillum* in roots of group H was significantly higher than that in roots of group L at tillering stage, which was 7.08 times of that in roots of group L, *Bacillus* in leaves of group H was significantly higher than that in leaves of group L, which was 3.61 times of that in leaves of group L, and *Buttiauxella* in group M was significantly higher than that of group L, which was 15.14 times that of group L.

As can be seen from Fig. 8 that the relative abundances of *Bradyrhizobium*, *Burkholderia* and *Anaeromyxobacter* in rice roots at maturity stage significantly increased due to the exploitation of rare earth, while the relative abundances of *Georgfuchsia*, *Pelomonas*, *Methylocystis*, *Streptomyces* and *Methylosinus* decreased significantly. The relative abundances of *Bradyrhizobium* in group H and M were 3017 and 11.89 times that of group L, respectively. The relative abundance of *Methylosinus* in group L was 5.24, 11.66, 6.20 and 6.34 times that of group H and M, respectively. The relative abundance of *Bradyrhizobium*, *Burkholderia*, *Geobacter*, *Denitisoma* and *Candidatus Koribacter* in rhizosphere increased, while relative abundance of *Gaiella* decreased. The relative abundance of *Geobacter*, *Candidatus koribacte*r and *Candidatus solibacte*r in rhizosphere of group H and M was 1.73, 2.68, 3.57 and 64.18 times that of group L, respectively. The relative abundance of gaiella in rhizosphere of group L was 601.5 and 601.5 times that of group H and M, respectively.

### Environmental factors affecting bacterial community of rhizosphere, root, phyllosphere and leaf in soil

**Figure 9:**
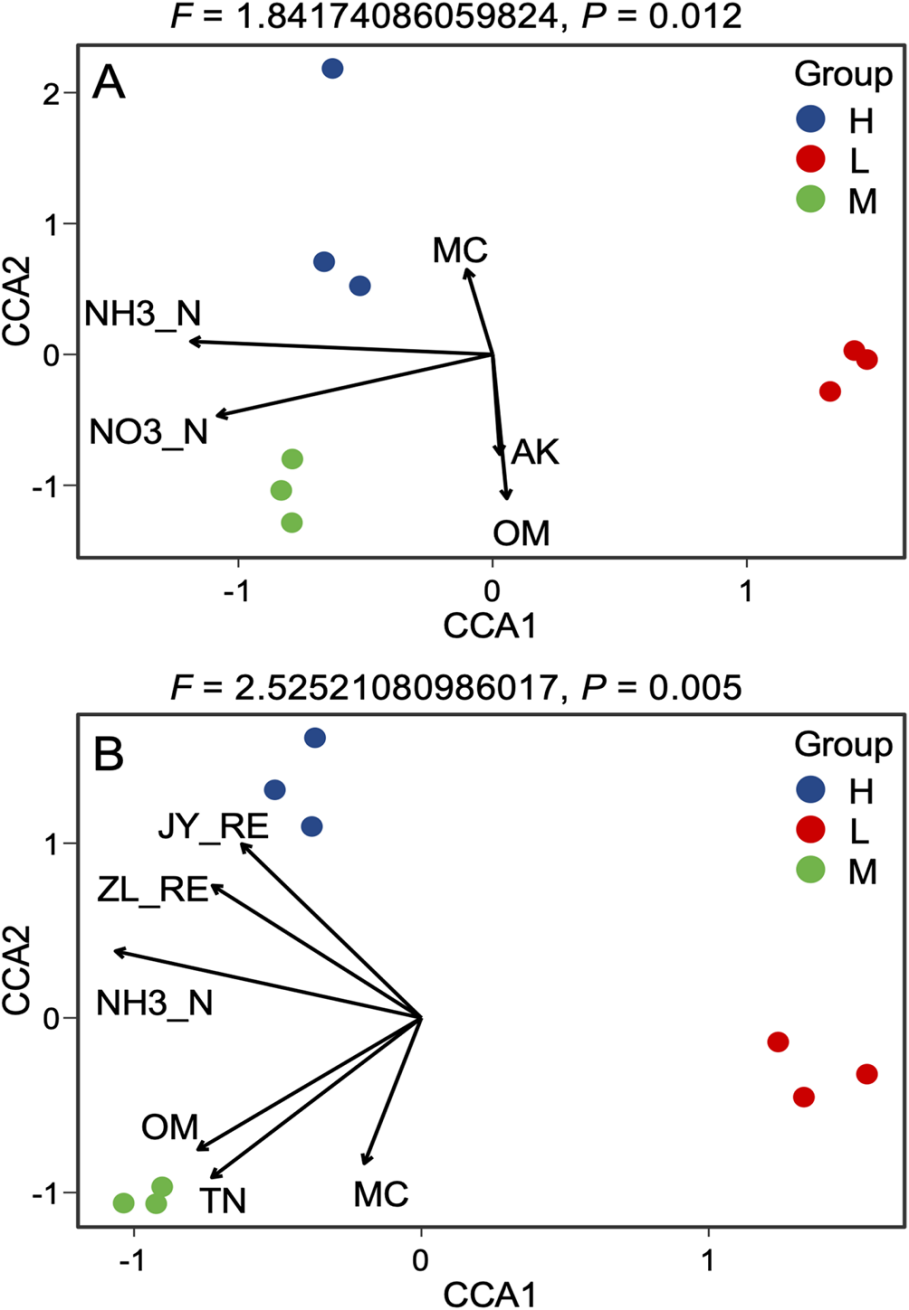
Canonical Correspondence Analysis (CCA) of rice bacterial community.

The bacterial community structure and construction process were affected by environmental factors. The relationship between bacterial community and environmental factors was analyzed by CCA. The results are shown in Fig. 9. Due to the differences in REE content and physicochemical properties of soil samples, each group of samples is obviously separated. Figure 9A shows the environmental factors affecting the three groups of sample H, M and L at the tillering stage of rice. It can be seen from the Fig. 9A that the main environmental factors are ammonia N, total N, available K and pH, etc.

Figure 9B shows the environmental factors affecting the three groups of sample H, M and L at the maturity stage of rice. It can be seen from the figure that the main environmental factors are the contents of rare earth elements in shoots, grains, ammonia nitrogen, organic matter and total nitrogen. Table 1 shows that Shannon, Richness, Pielou evenness of rice bacteria in rhizosphere, root, phyllosphere and leaf at tillering stage are significantly negatively correlated, highly negatively correlated or negatively correlated with REE content, total N, ammonia N, nitrate N, total P and available P in soil, rice roots, shoots, and highly positively correlated with pH. Chao.value was highly positively correlated with REE contents in roots, shoots of rice, and positively correlated with available K, organic matter and pH. As can be seen from Table 2 that the REE content in soil, rice roots, shoots, grains and soil physical and chemical properties are negatively correlated with the diversity, richness and evenness of bacteria in rhizosphere, root, phyllosphere and leaf at maturity period of rice, and highly negatively correlated with REE contents, ammonia N, nitrate N and pH in soil. As can be seen from Table 3 that the dry weight of rice roots, shoots, and grains are positively correlated with REE content, total N, ammonia N, nitrate N, total P, available P, available K, and organic matter in soil, rice roots, shoots, and negatively correlated with pH.

**Table 2:** Pearson correlation of rare earth element content, soil physical and chemical properties and α-diversity of rice bacteria at maturity stage.

	Shannon	Observed_richness	Pielou_evenness	Chao.value
Soil REE content	− 0.914	− 0.957	− 0.892	− 0.981
Root REE content	− 0.754	− 0.83	− 0.72	− 0.88
Shoot REE content	− 0.441	− 0.549	− 0.394	− 0.628
Grain REE content	− 0.589	− 0.685	− 0.546	− 0.752
Total N	− 0.695	− 0.6	− 0.731	− 0.52
Ammonia N	− 0.905	− 0.951	− 0.882	− 0.976
Nitrate nitrogen	− 0.999*	− 0.986	− 1.000**	− 0.964
Total P	− 0.786	− 0.703	− 0.816	− 0.63
Available P	− 0.797	− 0.715	− 0.827	− 0.644
Available K	− 0.704	− 0.61	− 0.739	− 0.53
Organic matter	− 0.777	− 0.693	− 0.809	− 0.62
pH	0.994	1.000**	0.987	0.994
Root weight	− 1.000**	− 0.993	− 0.998*	− 0.976
Leaf weight	− 0.993	− 1.000**	− 0.986	− 0.994
Grain weight	− 1.000**	− 0.991	− 0.999*	− 0.973

**At 0.01 level (two tailed), the correlation was significant, *At 0.05 level (two tailed), the correlation was significant, n = 3.

**Table 3:** Pearson correlation of rare earth element content, soil physical and chemical properties and dry weight of root, shoot and grain of rice.

	Nitrate nitrogen	Total P	Available P	Available K	Organic matter	pH	Total N	Ammonia N	Soil REE content	Root REE content	Shoot REE content	Grain REE content
Root weight	0.999*	0.783	0.794	0.701	0.775	− 0.994	0.693	0.907	0.915	0.757	0.444	0.592
Leaf weight	0.987	0.708	0.721	0.616	0.699	− 1.000**	0.607	0.948	0.955	0.826	0.542	0.679
Grain weight	0.999*	0.791	0.802	0.71	0.783	− 0.993	0.702	0.901	0.91	0.748	0.433	0.581

**At 0.01 level (two tailed), the correlation was significant, *At 0.05 level (two tailed), the correlation was significant, n = 3.

## Discussion

Previous studies have shown that lower concentrations of rare earth can promote the growth of crops, while higher concentrations can inhibit or even harm the growth of crops^12,13,31^. In this study, the shoots of rice tillers cultivated in soil of H and M in rare earth mining area were significantly more than those in non-mining area, especially rice cultivated in soil of M with medium rare earth content grew best. The dry weight of rice root, shoot and grain of soil H and M was significantly heavier than that of soil L. M was the heaviest, followed by H and L. The results are basically consistent with previous studies^3–5^. Due to extraction of ionic rare earth by (NH_4_)_2_SO_4_ leaching, ammonia N in mining area seriously exceeded the standard, pH value decreased, which caused dry weight of root, shoot, grain of cultivated rice in mining area to have significant negative correlation with nitrate nitrogen, and negative correlation with total nitrogen, ammonia nitrogen, and significant positive correlation with pH. A large number of studies have shown that there is a significant positive correlation between the REE content in crops and that in soil^2–5^. In this study, the content gradient of rare earth elements in soil is H > M > L, and the content gradient of rare earth elements in rice root, shoot and grain is H > M > L, which is basically consistent with the previous research results^3–5^.

Chao et al.^21^ considered that the REE content is the main factor affecting the microbial structure in rare earth mining area. Jiang et al.^32^ and Zhou et al.^33^ thought that high concentration of rare earth elements had inhibitory effect on bacteria. Yang et al.^34^ showed that when the concentration of rare earth ions reached a certain value, it had antibacterial effect; Some studies have shown that exogenous rare earth element Yttrium destroyed the structure of soil microbial community and significantly reduced OTUs, Ace, Chao1 and Shannon index^35^. Chen et al.^36^ found that rare earth mining seriously damaged the soil quality of the mining area, caused low diversity and evenness of microbial community. In this study, the OTU number of rice bacteria in rhizosphere, root and phyllosphere cultured in soil H and M of rare earth mining area was lower than that in soil L of non-rare earth mining area at tillering stage and maturity stage. In addition, the number of OTUs of bacteria in leaf of rice cultured in soil M was significantly lower than that in soil L. The richness and Chao indexes of bacteria in rhizosphere, root and phyllosphere of rice cultured in soil H and M were significantly lower than those in soil L at tillering stage and maturity stage. The Shannon index of H and M was significantly lower than that of L. The Pielou's evenness index of H and M were lower than that of L. It shows that the exploitation of rare earth reduces α-diversity of rice bacteria, which is consistent with the previous research conclusions of many scholars^19–21^. Mineral exploitation affects the REE content in soil, soil physical and chemical properties and rice growth, caused β-diversity of bacteria to be quite different.

Many studies have shown that soil heavy metals, physical and chemical properties, nutrient elements are important factors affecting microbial community structure^37–39^. Wei^40^ found that rare earth pollution affected the abundance of bacteria and fungi and changed their community structure. Wang^41^ found that the contents of bacteria, fungi and *Actinomycetes* decreased significantly, and the abundance and diversity of soil bacteria changed significantly. However, the dominant species of soil bacteria was Firmicutes, which was related to *Proteus*, *Actinomycetes*, *Bacteroidetes* and *Acidobacteria*. Firmicutes are affected by rare earth elements^3,5,41^. In this study, the abundance of Firmicutes in rice rhizosphere cultivated in soil H and M at tillering stage was significantly higher than that of soil L, and the abundance of Firmicutes in rice rhizosphere cultivated in soil H was significantly higher than that of soil M and L; The abundance of L was significantly higher than that of H and M; The abundance of Firmicutes in rice rhizosphere cultivated in soil L was significantly lower than that of soil H and M. It can be seen that Firmicutes (phylum) in soil–plant system are sensitive to rare earth elements, but the mechanism is very complex. Actinobacteria and Acidobacteria are greatly affected by soil physical and chemical properties. The former is directly proportional to pH value, while the latter is inversely proportional to pH value^3,5^. The pH value of rare earth mining area was reduced by mineral exploitation, and the abundance of Actinobacteria in rice rhizosphere of soil L was significantly higher than that in soil H and M, and the abundance of acidobacteria in rice rhizosphere of soil L was significantly lower than that in soil H and M.

The exploitation of rare earth minerals significantly reduced the diversity and evenness of soil–plant system microorganisms, and enhanced the resistance and growth of some bacteria, causing the formation of some dominant bacterial genera in rare earth mining area. For example, a large amount of acid mineral water was formed due to mineral exploitation, and the abundance of *Candida solibacter* and *Candida koribacter* in rice rhizosphere of soil M was higher^42^. Many dominant bacteria can promote the growth of rice, improve the yield of rice, and make the dry weight of rice root, shoots and grain be highly or extremely significantly negatively correlated to α-diversity of bacteria. Some scholars believe that the biological effect of rare earth ions may be similar to that of Ca ions because the radius of rare earth ions is close to that of Ca ions, rare earth ions may occupy or replace Ca ions in organisms^43,44^. Some researchers have explored the mechanism of the interaction between rare earth ions and microorganisms at the protein level. They believe that rare earth ions can affect the production of differential proteins by microorganisms, thus controlling the growth, reproduction and metabolism of microorganisms^45,46^. The differential proteins mainly include transporters, stress-related proteins, metabolism related proteins, etc. Chai et al.^47^ found that the effect of rare earth ions on promoting microbial reproduction was inhibited by chlorpromazine, indicating that the mechanism of La and Ce promoting bacterial growth may be related to calmodulin.

Soil environment, plants and microorganisms interact with each other, soil and plants affect bacteria, and bacteria also react on plants and soil^48^. Bacteria in soil–plant system can be divided into beneficial bacteria, neutral bacteria and harmful bacteria. Harmful bacteria endangers plant growth, and beneficial bacteria can play an important role in promoting plant growth, nutrient absorption and stress response^49–52^. Due to mineral exploitation, the REE content and ammonia nitrogen in soil increased significantly, the vegetation was destroyed, and the content of organic matter was low, which affected the ecological environment^53^. At tillering stage, *Ralstonia* abundance in root, rhizosphere, leaf of rice in soil H was high, accounting for 17.8%, 2.18% and 8.12% respectively. *Ralstonia* is a plant pathogenic bacteria, affecting the growth of rice^54^, which may be one of the reasons why the dry weight of root, shoot and grain of group H is lower than that of group M. The abundance of *Herbaspirillum*, *Azospirillum* and *Bacillus* in rice root, the abundance of *Azospirillum* in rice root and rhizosphere, The abundance of *Bacillus* in rice root, rhizosphere, leaf and phyllosphere of H is relatively high. Nitrogen fixation of *Herbaspirillum* and *Azospirillum* can promote plant growth, nutrient absorption and photosynthetic efficiency^55–57^. Bacillus has strong growth promoting effect on rice, potential antibacterial activity and strong antagonistic activity. It can produce iron carrier in iron limited medium and produce four kinds of secondary metabolites, causing damage to cell membrane of *Magnaporthe grisea*^58,59^. These beneficial bacteria may be an important reason why rice biomass in group H is significantly higher than that in group L.

Because of mineral exploitation, soil rare earth elements, total nitrogen, ammonia nitrogen and nitrate nitrogen in soil M were significantly higher than those in non-mining areas, pH decreased, soil acidity increased, forming a compound pollution of rare earth and ammonia nitrogen, causing bacterial species to be monotonous. At tillering stage, *Burkholderia* abundance in rice root and rhizosphere of M was very high, and *Acinetobacter*, *Buttiauxella* and *Comamonas* abundance in rice leaf was very high. *Burkholderia* is a microorganism with the ability to degrade ammonia nitrogen efficiently. It can transform ammonia nitrogen into substances needed by itself or beneficial to the environment. It has the functions of biological control, promoting plant growth, decomposing toxic substances and bioremediation. It is a potential genus for remediation of high ammonia nitrogen pollution in rare earth mine wasteland in South Jiangxi Province^60^. *Acinetobacter* can improve the resistance of plants to Cu, the ability to remove Ni and the ability to separate rare earth elements^61–63^. *Buttiauxella* could significantly improve the biomass and chlorophyll content of shoots and roots of plants treated with Cd^64^ and *Comamonas* also had the ability to degrade a variety of environmental pollutants^65^. These beneficial bacteria may be an important reason for the higher biomass of rice grew in soil M than that grew in soil H and L.

In the maturity stage, *Burkholderia*, *Bradyrhizobium*, *Ideonella* and *Pleomorphomonas* were found in rice rhizosphere of soil H. the abundance of *Geobacter* and *Denitisoma* was higher. The abundance of *Bradyrhizobium* in rice roots of soil M was especially high in maturity stage, and the abundance of *Geobacter* and *Candida_koribacter* in rice rhizosphere was high. *Bradyrhizobium Rhizobium* not only colonizes in legumes, but also in rice. The symbiosis between *Bradyrhizobium Rhizobium* and rice can achieve biological nitrogen fixation, which plays an important role in ensuring food security and ecological construction^66^. These bacteria may be another important reason why rice yield of soil H and M is significantly higher than that of soil L.

The contents of available P and ammonia nitrogen in L soil were significantly lower, and the relative abundances of *Pantoea* and *Acidovorax* in rice roots were higher at tillering stage. *Acidovorax* is the pathogen of brown streaked disease of rice^67^, which may be one of the reasons why rice yield cultivated in soil L is lower than that cultivated in soil H and M. *Pantoea* can promote the growth of alfalfa, promote the growth of wheat root and shoot, improve the availability of phosphorus in soil, and reduce the application of fertilizer^68^. The relative abundance of *Exiguobacterium* in rice phyllosphere of soil L is high. Some studies have shown that it has broad-spectrum antibacterial effect on Gram-positive and gram-negative food-borne pathogenic bacteria. It has antagonistic effect on a variety of plant pathogenic bacteria. It shows the ability of nitrogen fixation, phosphate solubilization and iron carrier generation^69,70^. The relative abundance of *Weissella* in leaves is high, which is beneficial bacteria^71^. The relative abundances of *Bradyrhizobium*, *Ideonella*, *Methylocystis*, *Streptomyces* and *Georgfuchsia* were higher in rice roots at maturity stage. Methylocystis can inhibit the growth of *Staphylococcus albicans* and *Bacillus subtilis* with spores, produce single cell protein, and have an important impact on the global C cycle^72^. *Streptomyces* has the potential of biocontrol against plant pathogenic bacteria^73^. *Georgfuchsia* is an aromatic compound degrading bacterium in aquifers^74^. These beneficial bacteria may be an important reason for the healthy growth of L rice under the condition of low soil fertility.

At tillering stage, Ralstonia was relatively abundant in rice root of soil H and moved vertically to rice leaf, resulting in higher relative abundance of Ralstonia in rice leaf; The relative abundance of *Ideonella* and *Bacillus* in rice rhizosphere was high, and moved horizontally to the root and leaf of rice, causing higher relative abundance in root and leaf; The relative abundance of *Burkholderia* and *Buttiauxella* in the rice rhizosphere of soil M was high, and moved horizontally to the root and leaf of rice, resulting in higher relative abundance of *Burkholderia* and *Buttiauxella* in the root and leaf; The relative abundance of *Exiguobacterium* in rice phyllosphere of soil L was high, and it moved horizontally into rice leaf, so that the relative abundance of *Exiguobacterium* in the rice leaf was also high. The relative abundance of *Azospira*, *Anaeromyces Obacter* and *Georgfuchsia* in the rice rhizosphere of soil H was higher in the maturity stage, and moved horizontally to the root, which made the relative abundance of *Azospira* higher in the root. The horizontal movement of these bacteria makes them play an active role in the process of rice growth and become an important reason for the healthy growth of rice^75^.

## Conclusion

1. The REE contents in rice roots, shoots and grains were significantly positively correlated with those in soil, causing the REE contents in all parts of rice in mining area to be significantly higher than those in non mining area. The dry weight of rice roots, shoots and grains was highly correlated with soil physical and chemical properties, nutrient elements and rare earth elements.
2. The exploitation of rare earth minerals inhibited α-diversity of bacteria in rhizosphere, root, phyllosphere and leaf of rice, significantly reduced the abundance index of OTU number, Chao1 and Ace index, decreased the diversity index of Shannon index, reduced the evenness index: Pielou's event index, etc., which caused β-diversity of bacterial to be different; Rare earth elements enhance the resistance and growth of some bacteria, and form some dominant bacteria genera in the rare earth mining area. They play an important role in promoting plant growth, nutrient absorption and stress response, which has become an important reason for the dry weight of rice cultivated in the soil of H and M in the mining area is significantly higher than that of soil L in non mining area.
3. Rare earth minerals reduce the diversity of bacteria, but form the dominant bacteria, such as *Burkholderia*, *Bacillus*, *Buttiauxella*, *Acinetobacter*, *Bradyrhizobium*, *Candida koribacter*, which can degrade the pollutants formed by rare earth mining, alleviate the compound pollution of rare earth and ammonia nitrogen, and have the functions of nitrogen fixation and resistance to rare earth stress; The content of soil available phosphorus in non mining area is low, forming the dominant bacteria of *Pantoea*, which has the function of improving soil phosphorus availability.
4. Rare earth elements and physical and chemical properties of soil affect the community structure of bacteria in rhizosphere and phyllosphere of rice, promote the parallel movement of some bacteria in rhizosphere, root, phyllosphere and leaf of rice, promote the construction of community structure of bacteria in rhizosphere and phyllosphere of rice, give full play to the growth promoting function of Endophytes, and promote the growth of rice.

## Materials and methods

### Soil collection and material preparation

**Figure 10:**
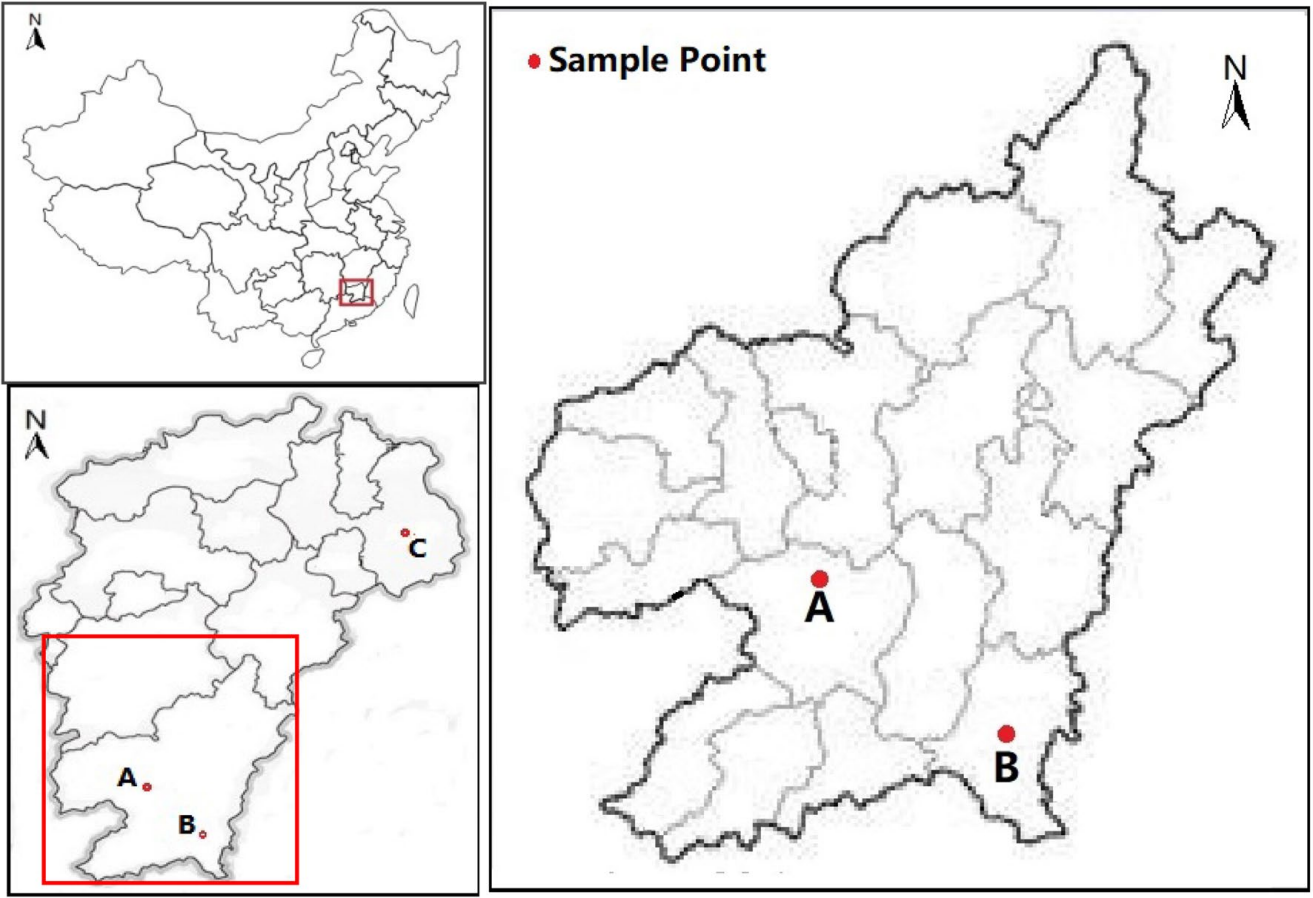
Distribution map of Sampling Points in Southern Jiangxi of China. Notes: “A” stands for Longshe Village, Jiading Town of Xinfeng County; “B” stands for Shipai Village, Wenfeng Town of Xunwu County. (The map is drawn by arcGIS 10.7, and URL is: https://www.esrichina.com.cn/ArcGIS/arcgis10_7.html/onegreen.net/maps/m/jiangxi.htm).

Figure 10 shows the sampling point of rare earth mining area: 1500 m away from REE mining area (latitude and longitude is 114°59′36″E, 25°23′57″N) in Longshe village, Jiading town, Xinfeng County, southern Jiangxi Province; 1500 m away from REE mining area (latitude and longitude is 115°42′6″E, 24°52′9″N) in Shipai village, Wenfeng Township, Xunwu County, southern Jiangxi Province and the sampling point of non REE mining area is located in Choukou village, Maojialing Township, Xinzhou District, Shangrao City of Jiangxi Province(longitude and latitude is 117°57′46″E, 28°25′1″N). 50 kg of 20 cm topsoil were collected at three sampling points respectively. Air dry the soil sample naturally, remove the grass roots, litter, stones and other debris, and sift through 100 mesh for standby. The prepared test soil was loaded into a 26 cm high and 22 cm diameter rigid PVC bucket with 5 kg of soil, 5.4 g of urea (containing 46.6% n), 0.6 g of potassium chloride (containing 62.9% K_2_O) and 1.00 g of superphosphate (containing 14.0% P_2_O_5_). The soil was mixed with N, P and K fertilizers, and rice was planted after a week of submergence.

### Rice pot experiment and sample collection

Rice variety Jiangzao 361 was purchased from Jiangxi Keyuan Seed Industry Co., Ltd. The whole growth period of this variety is 110.2 days. After participating in the regional test of rice in Jiangxi Province from 2013 to 2014, it is widely planted in Jiangxi Province. The seeds were disinfected with 10% H_2_O_2_ solution for 15 min, then washed with water for many times.

After germination, the seeds were sown in the soil without rare earth pollution for seedling raising. Four weeks later, the rice seedlings were transplanted into PVC buckets with culture soil prepared. Two seedlings were transplanted into each barrel. The pot experiment was carried out in the greenhouse. The daily illumination, duration of illumination, temperature and humidity of the greenhouse were adjusted to be close to that of the outdoor. According to the growth characteristics of Jiangzao 361 rice variety, the pot-experiment rice was managed in combination with the experience and methods of rice planting management in Jiangxi Province. According to the REE content in soil, the samples were labeled as H, M and L. The soil with highest REE content was H, followed by M and L. H and M are both the soils from rare earth mining area. The pot-experiment rice was labeled with H1, H2, H3, M1, M2, M3, L1, L2, L3 respectively because three replicates were made for each sample.

Rhizosphere soil, rice roots, shoots were collected at tillering stage, and rhizosphere soil and rice roots were collected at maturity stage, which was used for molecular experiments.

When the rice matured, the roots, shoots and millet of rice were harvested respectively, washed with deionized water, then placed in the ventilated room and dried naturally until constant weight and weighed. Husk the millet and collect the unpolished rice. The unpolished rice, shoot and root were ground into powder and stored at room temperature for analysis.

### Chemical analysis

The soil physical and chemical properties and nutrient elements are tested by the relevant detection methods in the Agricultural Industry Standard of the People's Republic of China (2006). Soil pH determination: NY/T 1121.2-2006, determination of total P in soil: alkali soluble molybdenum antimony anti spectrophotometry HJ632-2011, determination of soil available phosphorus: sodium bicarbonate extraction molybdenum antimony anti spectrophotometry HJ704-2014, determination of soil ammonia nitrogen, nitrite nitrogen and nitrate nitrogen: potassium chloride solution extraction spectrophotometry HJ634-2012, determination of soil available K and slow acting potassium content: NY/t889-2004, Determination of soil organic matter: NY/T 1121.6-2006. The test results can been seen in Table 4.

**Table 4:** Soil physical and chemical properties, nutrient elements and REE content (mg/kg).

Sample name	pH	Total N	Ammonia N	Nitrate N	Total P	Available P	Available K	Organic matter (%)	REE content
H1	5.28	1351.96	308.37	29.7	338.9	13.59	70.82	2.81	916.56
L1	6.58	1425.36	198.08	24.98	114.49	4.03	77.32	4.58	140.13
M1	4.97	2624.05	311.43	34.27	1141.24	87.1	81.5	5.67	824.05
H2	5.52	1425.31	124.37	29.33	180.6	9.9	83.34	4.11	805.64
L2	6.63	1551.15	38.55	25.33	112.93	1.44	85.49	4.05	110.33
M2	5.2	2839.02	113.75	31.65	1220.12	106.47	111.49	5.34	735.09

H1, M1 and L1 are rice and soil samples in tillering stage, H2, M2 and L2 are rice and soil samples in maturity stage, the same below.

0.25 g of soil sample and 5 ml aqua regia (HNO_3_:HCl = 3:1) is added to a 100 ml digestion tube (three replicates for each sample), then balance overnight at room temperature, and heat to 120 °C for 12 h, then warm to 140 °C until the soil color turns white, and the digestion procedure refers to Ref.^3^. After digestion, the sample was cooled in a fume hood and diluted to 50 ml with ultrapure water. After filtration with 0.45 μm membrane, the contents of 15 rare earth elements (Y, La, Ce, Pr, Nd, Sm, Eu, Gd, Tb, Dy, Ho, Er, Tm, Yb, Lu) were determined by ICPMS-2030 (Shimadzu Corporation, Shimadzu Institute, Kyoto, Japan). The quality was monitored by the standard reference substance GBW07405 of the national standard substance research center. The test results are shown in Table 1.

0.2 g of crushed unpolished rice, 0.1 g of rice shoot, and 0.1 g of rice root was added to a 50-ml polyethylene centrifuge tube (three replicates for each sample) respectively, soaked in 3 ml of superior pure nitric acid overnight, and digested in a microwave digestion oven (Mars, Matthew Inc., USA), The whole process of digestion was controlled by GBW07603 (GSV-2), a national first-class reference material (Institute of Geophysical and Geochemical Exploration, Ministry of Geology and Mineral Resources of China). The digestion solution was diluted to 50 ml with ultrapure water, filtered with 0.45 μm membrane. The contents of 15 rare earth elements were determined by ICPMS-2030.

### Pretreatment and DNA extraction of soil and plant samples

The loose and massive soil of rice root samples were removed, and then immersed in 15 ml PBS buffer containing Silwet L-77 (pH 7.0, 0.02% Silwet L-77) and shook in shaking table for 10 min. Then the suspension obtained was poured into a sterile centrifuge tube and centrifuged at 6000*g* for 5 min. The precipitates were taken as rhizosphere soil samples and stored at − 80 °C for subsequent DNA extraction. After shaking, the roots were washed in 75% ethanol for 10 min, 2.5% sodium hypochlorite for 10 min, and finally washed in sterile water for 5 times to remove the residual microorganisms on the root surface (scanning electron microscope was used to check whether it was clean). The cleaned roots were defined as root samples^76,77^. The root samples were ground in PBS with sterile mortar and pestle, washed into the centrifuge tube and left for 30 min. The supernatant was centrifuged at 6000*g* for 5 min, and the cell particles obtained (endophyte samples) were stored at − 80 °C for subsequent DNA extraction.

The leaves were placed in a sterile conical flask (250 ml volume) with 100 ml PBS (pH 7.0, 0.02% Silwet L-77) and stirred violently for 30 min. the leaves were then ultrasonically washed in water bath for 10 min. The supernatant (phyllobacteria sample) was concentrated at 0.22 μM nitrocellulose membrane filter. Before extraction of DNA, the membrane was stored at − 80 °C. Then the leaves were washed in 75% ethanol for 3 min, washed in 2.5% sodium hypochlorite for 5 min, and washed with sterile water for 5 times. Then the leaves were ground in PBS with sterile mortar and pestle, washed into the centrifuge tube and left for 30 min. The supernatant was transferred to a new centrifuge tube and centrifuged at 6000*g* for 5 min. The extracted cell microspheres (endophyte samples) were stored at − 80 °C before DNA extraction.

The soil samples of rice rhizosphere were pretreated with propidium monoazide (PMA) to remove the DNA interference of dead microorganisms^78^. Then, the DNA of bacteria sample in rhizosphere soil, root, phyllosphere and leaf was extracted by FastDNA™SPIN Kit for Soil (MP Biomedicals LLC, USA). The DNA concentration and purity were determined by nanodrop 2000, and then stored at − 20 °C in refrigerator for 16S rRNA gene analysis.

### Amplification and sequencing of 16S rRNA gene

The V4 region of 16S rRNA was amplified by bacterial specific primers 515F (5′-GTGCCAGCMGCCGCGGTAA-3′) and 806R (5′-GGACTACHVGGGTWTCTAAT-3′). Phusion high fidelity enzyme (high fidelity PCR master with GC buffer) was used in PCR. The process was as follows: pre-denaturation for 5 min in 95 °C, denaturation for 30 s in 94 °C, annealing for 35 s in 55 °C, extension for 30 s in 72 °C, the above process is carried out 30 times, then extension for 8 min in 72 °C. The gel Life Technology (USA) was used for recovery and purification. After being qualified by gel electrophoresis, the accurate double stranded DNA concentration was quantified by Qubit3.0 (Life Technology, USA).

The NEB kit was used to construct the library, the standard database building process of DNA Library Prep Kit for Illumina. NEBNext Ultra (NEB#e7370S/L) was adopted. The length distribution and concentration of the constructed library was detected by aglent2100 and qPCR, respectively. The qualified library was sequenced by Illumina hiseq2500 (PE250 reads). The sequencing samples included soil and plant samples (rhizosphere soil samples, phyllospheric bacteria samples, endophytic bacteria samples in leaves and endophytic bacteria samples in roots) at tillering stage and maturity stage. The data obtained after sequencing were used for quality control.

The 16S rRNA gene sequences obtained by high-throughput sequencing were pretreated and quality controlled by QIIME2 software. Quality control adopted the parameters recommended by Bokulich et al.^79^ According to the sequence similarity, they were classified into multiple OTUs. Those gene sequences with similarity level of 0.03 (97% sequence similarity, approximately equal to species level) were selected for subsequent analysis, and the OTU representative sequence was annotated. The reference sequence from Silva database (db128 version) is used, the command of mothur software (classify. SEQS) is used for annotation, and RDP algorithm is used for annotation algorithm.

### Statistical analysis

QIIME2 software was used to analyze the a-diversity (richness index, Shannon index, etc.) and β-diversity (based on UniFrac and Bray Curtis equidistance matrix), vegan package (v.3.4.1) of R software^80,81^ is used for NMDS visual analysis to understand clustering characteristics of β-diversity. Nonparametric multivariate statistical algorithms such as PERMANOVA.ANOSIM in vegan package (v.3.4.1) of R software^80,81^ were used to analyze the effects of soil basic physical and chemical properties, nutrient factors and REE content on rice. Pearson test, canonical correspondence analysis (CCA) and variation partitioning analysis (VPA) based on CCA were used to analyze the contribution of environmental variables to bacterial community. Origin9.0 is used to draw Heatmap, Software of Metasee and Qiime are used for data visualization^6,21^.

### Statement on guidelines as experimental research and field studies on rice

Experimental research and field studies on rice (cultivated rice and wild rice), including the collection of rice comply with relevant institutional, national, and international guidelines and legislation, and this studies comply with local and national regulations. The measurement process of microorganism and rare earth content in different parts of rice will not affect the local soil microorganism and ecological environment, etc. During the process of experiment, aseptic sampling was carried out to avoid contamination, and the research was evaluated and agreed by the environmental protection authorities of the local government.

The experimental group and control group have been tested at the same time, and the whole experimental process of the research is safe, which is based on references as below.

1. The methods of soil collection and material preparation are mainly from Refs.^2–5^.
2. The methods of potted rice and sample collection are mainly from Refs.^3–5^.
3. The soil physical and chemical properties and nutrient elements were detected by the relevant detection methods in the Agricultural Industry Standard of the People's Republic of China (2006).
4. The determination methods of rare earth elements in soil and rice are mainly from Refs.^2–5^.
5. The pretreatment and DNA extraction of soil and plant samples are mainly from Refs.^76–78^.
6. Amplification and sequencing of 16S r RNA gene is mainly from Refs.^79,34^.
7. Statistical analysis is mainly from Refs.^6,21^.
